# Comorbid tobacco and other substance use and symptoms of anxiety and depression among hospitalised orthopaedic trauma patients

**DOI:** 10.1186/s12888-019-2021-y

**Published:** 2019-01-17

**Authors:** Sam McCrabb, Amanda L. Baker, John Attia, Zsolt J. Balogh, Natalie Lott, Kerrin Palazzi, Justine Naylor, Ian A. Harris, Christopher M. Doran, Johnson George, Luke Wolfenden, Eliza Skelton, Billie Bonevski

**Affiliations:** 1 School of Medicine and Public Health, Faculty of Health and Medicine, University of Newcastle, 1 University Drive, Callaghan, NSW 2308 Australia; 2 Hunter Medical Research Institute, University of Newcastle, New Lambton, New South Wales 2305 Australia; 3 Department of General Medicine, John Hunter Hospital, New Lambton Heights, New South Wales 2305 Australia; 4 Department of Traumatology, John Hunter Hospital, New Lambton, New South Wales 2305 Australia; 5 Whitlam Orthopaedic Research Centre, Ingham Institute for Applied Medical Research, Liverpool Hospital, Liverpool, New South Wales 2170 Australia; 6 South Western Sydney Clinical School, Faculty of Medicine, University of New South Wales, Liverpool, New South Wales 2170 Australia; 7 School of Human, Health and Social Sciences, Central Queensland University, Brisbane, Queensland 4000 Australia; 8 Centre for Medicine Use and Safety, Monash University, Parkville, Victoria 3052 Australia; 9 Hunter New England Population Health, Wallsend, NSW 2287 Australia

**Keywords:** Smoking, Alcohol, Cannabis, Anxiety, Depression, Comorbidities

## Abstract

**Background:**

No study has examined the prevalence of tobacco, other substance use, and symptoms of anxiety and depression, and rates of comorbidities among the orthopaedic trauma population, despite the impact they have on recovery from surgery. This study aims to 1) describe the rates of symptoms and substance use; 2) compare rates of symptoms and substance use among smokers versus non-smokers; and 3) examine the relationship between symptoms and substance use with smoking status.

**Methods:**

A cross-sectional survey of orthopaedic trauma patients was conducted in two Australian public hospitals. Demographic characteristics, smoking status, alcohol consumption, recent cannabis use, and symptoms of anxiety and/or depression were examined. Differences between current and non-smokers were compared using Pearson Chi^2^ tests. Multivariate logistic regression explored variables related to tobacco smoking.

**Results:**

Eight hundred nineteen patients participated. Over one-fifth (21.8%) identified as a current smoker, half (51.8%) reported consuming alcohol at hazardous levels in the last 12 months, and about 10% stated that they had used cannabis in the last 30 days (9.7%), or experienced symptoms of either anxiety (12.4%), or depression (12.9%) in the last two weeks. Over one-fifth of current tobacco smokers (21.8%) reported drinking heavily in the last 12 months and using cannabis recently. Males, with a lower educational attainment, who were unmarried, had used cannabis recently, and report drinking heavily were more likely to be current smokers.

**Conclusions:**

Health behaviour interventions addressing comorbidities are warranted among the orthopaedic trauma population given the high rate of comorbidity and impact these may have on recovery.

**Electronic supplementary material:**

The online version of this article (10.1186/s12888-019-2021-y) contains supplementary material, which is available to authorized users.

## Background

The impact of tobacco smoking on recovery from orthopaedic trauma surgery is well known, with cigarettes linked to increased risk of post-operative infection, [1–3] wound and flap necrosis, [4] and a decrease in the tensile strength of wounds [5]. Despite this, rates of tobacco smoking among orthopaedic trauma patients remain higher than that in the general population (37% vs. 14.5% in 2014) [6, 7].

When treating tobacco addiction, it is important to identify factors that may undermine quit attempts. Comorbidity, that is, having more than one co-occurring physical or mental health condition at any one time, [8] is known to complicate smoking cessation treatment [9]. Rates of symptoms of anxiety and depression have been found to be higher in orthopaedic trauma patients (39 and 30.5% respectively) [10] when compared to the general population, [7] and rates of symptoms of anxiety and depression continue to remain high up to two years post-hospital discharge (29.4 and 37.6%, respectively) [11]. Previous research also indicates that rates of hazardous alcohol and recent cannabis use are high in the orthopaedic trauma population [12]. In addition, orthopaedic trauma patients who are readmitted to hospital are more likely to use tobacco, alcohol and other drugs, indicating the need to address the comorbidities within the orthopaedic trauma population [13]. As far as the authors are aware, rates of smoking, alcohol and cannabis use, and symptoms of depression and anxiety have yet to be assessed in the one study among orthopaedic trauma patients.

Guidelines recommend provision of smoking cessation care and nicotine replacement therapy (NRT) during the inpatient stay [14, 15]. Research suggests that concurrent treatment of co-occurring tobacco and other substance use is more effective than delayed treatment [16]. Concurrent treatment of alcohol use and depression, [17] and depressive symptoms and smoking [18] have been found to be effective. Further, treating tobacco addiction has a positive impact on rates of use of other drugs, with tobacco cessation related to long term reduction or abstinence from other drugs [19]. Thus, if comorbid smoking, alcohol use, cannabis use, and symptoms of depression and anxiety are found to be high among a cohort of hospitalised orthopaeic trauma patients, then there would be implications for the treatment of tobacco use in this setting.

We hypothesise that addressing comorbid health conditions of tobacco smoking (such as other substance use or mental ill-health) when providing smoking cessation care in this setting may: a) improve the health outcome of patients faster, given the negative health impact substance use and mental ill-health can have on recovery; [1–3] and b) decrease health care costs and lengths of hospital admissions, given continued substance use may result in readmission or longer hospital stays, [20] and that are readmitted to hospital are more likely to use substances [13]**.**

Thus, this study aims to characterise an orthopaedic trauma population, and build on prior research in this population [6, 12]. Specifically, this study aims to:

1. describe the rates of symptoms of anxiety, depression, and substance use (alcohol and cannabis) among patients admitted for an orthopaedic trauma;
2. compare rates of symptoms of anxiety, depression, and alcohol and cannabis use among smokers versus non-smokers; and.
3. examine the relationship between comorbid symptoms of anxiety and depression, alcohol and recent cannabis use with current tobacco use.

## Methods

### Study design and setting

An online cross sectional survey of orthopaedic trauma inpatients was conducted at two Level 1 Trauma public hospitals in New Souths Wales, Australia. Surveys were conducted between April 2015 and September 2016 during the individual’s hospital admission.

### Participants

Patients were included if they were: admitted to hospital with a fracture (skull and c-spinal fractures not included); aged 18–80 years; and were able to read and comprehend written English. Patients judged incapable of providing consent (such as those documented to have post-traumatic amnesia, or with a traumatic brain injury) were not approached to take part. All participants provided verbal informed consent prior to commencing the online survey.

Individuals were approached during admission by a research assistant to participate in an online health survey of orthopaedic trauma patients. Research assistants were provided with a list of new orthopaedic trauma admissions each day. Survey numbers were assigned to patients to de-identify their demographic and clinical information from their study results. Patients completed the survey using an internet enabled tablet device during hospital admission. If an individual was too unwell to be seen or was busy with medical staff, they were approached the following day.

### Measurements

Existing validated survey items were used, where possible; [21–27] relevant survey questions are presented in Additional file 1: Supplement 1. Average time to completion of the questionnaire was 15 min. Demographics, current smoking status, alcohol use, cannabis use, and symptoms of anxiety or depression were measured.

*Demographic characteristics.* Participants were asked to report: gender; age; country of birth; indigenous status; marital status; education; main source of income; household income; and health insurance type.

*Smoking status and smoking related variables.* Based on previous research; [21] daily, occasional, ex- and non-smoker status was determined. For participants who identified as current smokers, specific questions related to tobacco consumption were asked, [22, 23] with the Heaviness of Smoking Index (HSI) used to determine nicotine dependence (0–2 low, 3–4 moderate, and 5–6 high) [22, 28–30].

*Alcohol use.* Alcohol consumption in the last 12 months was measured using the 3-item Alcohol Use Disorder Identification Test (AUDIT-C) [24] to screen for hazardous use. Scoring for the AUDIT-C ranges from 0 to 12 with cut-offs of three for females (sensitivity 66–73%, specificity 91–94%) [31, 32] and four for males used to indicate a hazardous drinker (sensitivity 86%, specificity 72–89%) [24, 32]. For this study, a non-drinker was categorised as an individual who scored a zero on the AUDIT-C. A score of 1–3 for males or 1–2 for females was regarded as a non- hazardous drinker. Scores above this indicated hazardous drinking.

*Cannabis use.* Cannabis use during the previous 30 days was measured using an item based on questions asked in the Opiate Treatment Index (OTI) [25].

*Anxiety symptoms.* The Generalised Anxiety Disorder two item (GAD-2) screening tool [26] was used to estimate symptoms of anxiety in the last two weeks. Scoring for the GAD-2 ranged from 0 to 6, with a cut off score of three or more indicating anxious symptoms. Specificity for this item ranges from 81% to 88% and sensitivity ranges from 59% to 86% [26].

*Depression symptoms.* The Patient Health Questionnaire (PHQ-2), [27] a brief 2-item validated scale was used to estimate rates of symptoms of depression in the last two weeks. Scoring for the PHQ-2 ranges from 0 to 6, with a cut off score of three or more indicating depressive symptoms. Specificity for this item is 86% and sensitivity is 79% for any depressive disorder [27].

### Analysis

All data were stored on secure servers at the University of Newcastle and exported into STATA v13 (StataCorp LP., College Station, TX, USA) for analyses.

Descriptive statistics of participant demographics are presented as numbers and percentages for categorical variables and means (standard deviation; SD) for continuous variables. Rates of smoking were compared among demographic and comorbid groups using Pearson Chi-squared test (categorical) or independent t-test (continuous). Further, a binary logistic regression model was used to examine the associations between demographic characteristics, substance use, and rates of symptoms of anxiety and depression and current tobacco use (compared to non-tobacco use). Variables included in the model were selected a priori and included factors known to be associated with tobacco smoking in medically ill and general populations: age, gender, country of birth, education, marital status, household income, [33–35] anxiety, depression, [8, 36] alcohol, and cannabis use. Crude and adjusted odds ratios (OR) with 95% confidence intervals (CI) and *p*-values are presented. Significance was determined at *p* < 0.05. Cronbach alpha statistics are provided for established survey items (AUDIT-C, PHQ-2, and GAD-2) in Additional file 2: Supplement 2.

## Results

Of 1128 orthopaedic trauma patients approached, 819 agreed to participate in the survey (72 refused, 103 were too ill to participate, and 134 were not eligible; response rate of 73%). During survey completion, some individuals dropped out due to fatigue. A total of 805 individuals completed the survey (completion rate 98.2%). Of the 14 participants who did not complete the survey, five were male (nine female), and the average age was 53.6 years (SD 13.4). Two of 13 participants had identified as current tobacco smokers (one participant had dropped out prior to reporting smoking status). Results from this population group has already indicated that interest in quitting smoking is high but that provision of smoking cessation care during admission was low [37]. Six participants reported as non-cannabis users, and of the six who reported their alcohol consumption, two were non-drinkers, two were non- hazardous drinkers and two were hazardous drinkers (the remaining eight participants had dropped out prior to reporting their alcohol or cannabis consumption). The overall Cronbach alpha statistics for AUDIT-C, PHQ-2, GAD-2, were acceptable (ranging from 0.7–0.9), [38] and did not indicate the need to remove any sub-measures.

*Demographic characteristics.* Table 1 contains a summary of patient demographic information. The mean age was 50.6 years (SD 17.7), the majority of participants were male (59.7%), born in Australia (84.1%), and identified as non-Indigenous (94.6%).

**Table 1:** Sociodemographic characteristics of the sample

Characteristic		Total (*n* = 819)^a^ n (%)
Gender	Male	489 (59.7%)
	Female	330 (40.3%)
	Missing	0
Age	Mean (SD)	50.6 (17.7)
	Missing	0
Country of birth	Australian	688 (84.1%)
	Other	130 (15.9%)
	Missing	1
Indigenous status	Aboriginal	39 (4.8%)
	Torres Strait Islander	4 (0.5%)
	Both Aboriginal and Torres Strait Islander	1 (0.1%)
	Neither	774 (94.6%)
	Missing	1
Marital Status	Married/ Defacto/Living with Partner	471 (57.6%)
	Single/Widowed/ Separated/Divorced	347 (42.4%)
	Missing	1
Education	No formal education/Primary school/ High school 7–10	262 (32.0%)
	High school 11–12	131 (16.0%)
	TAFE or trade/University	425 (52.0%)
	Missing	1
Main source of income	Paid employment (either full or part time)	427 (52.2%)
	Government pension or benefit	293 (35.8%)
	Family member	29 (3.6%)
	Savings or retirement funds	37 (4.5%)
	Other	32 (3.9%)
	Missing	1
Household Income	<$50,000 per year	293 (36.0%)
	$51,000 - $100,000 per year	221 (27.1%)
	Over $100,000	101 (12.4%)
	Prefer not to state	200 (24.5%)
	Missing	4
Insurance type	Private health insurance/Department of Veteran’s Affairs white or gold card	349 (42.8%)
	Health care concession card	243 (29.8%)
	None of these	223 (27.4%)
	Missing	4

^a^Some respondents may have dropped out during survey completion due to fatigue

*Rates of anxiety or depressive symptoms and substance use.* Table 2 reports the rates of anxiety or depressive symptoms and substance use for the total sample.

**Table 2:** Rates of tobacco use, alcohol consumption, recent cannabis use, symptoms of depression (PHQ-2) and anxiety (GAD-2) among the sample

Characteristic		Total (*n* = 819)^a^ n (%)
Smoking status	Daily smoker	157 (19.6%)
	Occasional Smoker	18 (2.2%)
	Ex-smoker	235 (29.3%)
	Non-smoker	393 (48.9%)
	Missing	16
AUDIT-C	Hazardous drinker	412 (51.8%)
	Non-hazardous drinker	198 (24.9%)
	Non-drinker	185 (23.3%)
	Missing	24
Cannabis use in last 30 days	Yes	79 (9.7%)
	No	732 (90.3%)
	Missing	8
Mix tobacco with cannabis?	Yes, every time I smoke	49 (6.1%)
	Yes, sometimes	33 (4.1%)
	No, never	728 (89.9%)
	Missing	9
Depression	Above threshold for depression marker	104 (12.9%)
	Below threshold for depression marker	705 (87.1%)
	Missing	10
Anxiety	Above threshold for anxiety marker	100 (12.4%)
	Below threshold for anxiety marker	708 (87.6%)
	Missing	11
Either Depression or Anxiety	Above threshold	153 (18.9%)
	Below threshold	655 (81.1%)
	Missing	11
Both Depression and Anxiety	Above threshold	51 (6.3%)
	Below threshold	757 (93.7%)
	Missing	10

^a^Total n may differ as some respondents may have dropped out during survey completion due to fatigue

*Differences between current and non-smokers.* Table 3 shows the substance use differences between current and non-smokers. Rates of substance use were significantly higher in smokers than non-smokers, as were rates of anxiety and depression.

**Table 3:** Rates of tobacco use, alcohol consumption (AUDIT-C), recent cannabis use, symptoms of depression (PHQ-2), and anxiety (GAD-2), by smoking status

Characteristic		Smokers^a^ (*n* = 175) n (%)	Non-smokers^a^ (*n* = 628) n (%)	*p*-value
Smoking status	Daily smoker	157 (89.7%)	–	
	Occasional Smoker	18 (10.3%)	–	
	Ex-smoker	–	235 (37.4%)	
	Non-smoker	–	393 (62.6%)	
	Missing	0	0	
Alcohol consumption	Non-drinker	26 (15.3%)	159 (26.1%)	< 0.001
	Non- hazardous drinker	25 (14.7%)	165 (27.1%)	
	Hazardous drinker	119 (70.0%)	286 (46.9%)	
	Missing	5	18	
Cannabis use in last 30 days	No	122 (70.1%)	597 (96.0%)	< 0.001
	Yes	52 (29.9%)	25 (4.0%)	
	Missing	1	6	
Mix tobacco with cannabis?	No	119 (68.4%)	596 (96%)	< 0.001
	Yes, every time I smoke	40 (23.0%)	9 (1.5%)	
	Yes, sometimes	15 (8.6%)	16 (2.6%)	
	Missing	1	7	
Depression	Below threshold for depression marker	141 (81.0%)	550 (88.7%)	0.008
	Above threshold for depression marker	33 (19.0%)	70 (11.3%)	
	Missing	1	8	
Anxiety	Below threshold for anxiety marker	140 (80.9%)	554 (89.4%)	0.003
	Above threshold for anxiety marker	33 (19.1%)	66 (10.7%)	
	Missing	2	8	
Either depression or anxiety	Below threshold	125 (72.3%)	516 (83.2%)	0.001
	Above threshold	48 (27.6%)	104 (16.8%)	
	Missing	2	8	
Both depression and anxiety	Below threshold	155 (89.6%)	588 (94.8%)	0.013
	Above threshold	18 (10.4%)	32 (5.2%)	
	Missing	2	8	

^a^Some respondents may have dropped out during survey completion due to fatigue

*Rates of concurrent alcohol and cannabis usage for current tobacco smokers.* Almost a quarter of current tobacco smokers (21.8%) reported drinking heavily in the last 12 months and using cannabis recently (Table 4). A further 48.2% of current tobacco smokers had reported drinking heavily in the last 12 months, but had not used cannabis in the last 30 days.

**Table 4:** Concurrent use of alcohol (AUDIT-C), cannabis and tobacco according to smoking status

Cannabis use in the last 30 days	Alcohol consumption	Smoking status	
		Smoker *n* = 170 (%)	Non-smoker *n* = 610 (%)
No	Non-drinker	21 (12.4%)	156 (25.6%)
	Non- hazardous drinker	15 (8.8%)	160 (26.2%)
	Hazardous drinker	82 (48.2%)	270 (44.3%)
Yes	Non-drinker	5 (2.9%)	3 (0.5%)
	Non- hazardous drinker	10 (5.9%)	5 (0.8%)
	Hazardous drinker	37 (2 1.8%)	16 (2.6%)

*Relationship between symptoms of anxiety, depression, and substance use with current smoking status.* Table 5 shows that gender, education level, marital status, cannabis use and level of alcohol use were found to be associated with current tobacco smoking. Males had greater odds of being current tobacco users (OR 1.79 95% CI 1.14, 2.82; *p* = 0.01). The odds of being a current smoker were 2.2 times higher in individuals who had an education level of year 10 or less, when compared to those with a tertiary education (*p* < 0.001). Relationship status was found to be significantly associated with current tobacco use, with the odds of being a current smoker 1.5 times higher for single/widowed/divorced/separated individual when compared to an individual in a married/defacto relationship (*p* = 0.05).

**Table 5:** Crude and adjusted logistic regression of smoking status, with age, gender, marital status, income, depression (PHQ-2), anxiety (GAD-2), cannabis use and alcohol use (AUDIT-C) as explanatory variables

		Crude		Adjusted^a^	
	Smoker *n* = 175 (%)	Odds Ratio (95% CI)	*P*-value	Odds Ratio (95% CI)	*P*-value
Age		0.98 (0.97, 0.99)	< 0.001	0.99 (0.98, 1.00)	0.067
Gender			< 0.001		0.012
Female	46 (14.3%)	Ref.		Ref.	
Male	129 (26.8%)	2.20 (1.52, 3.20)		1.79 (1.14, 2.82)	
Country of birth			0.044		0.673
Other	19 (15.0%)	Ref.		Ref.	
Australian	156 (23.1%)	1.71 (1.01, 2.87)		1.13 (0.63, 2.03)	
Education			< 0.001		0.002
TAFE Trade/University	68 (16.2%)	Ref.		Ref.	
High School 11–12	28 (22.2%)	1.47 (0.90, 2.42)	0.123	1.16 (0.65, 2.08)	0.606
No Formal/Primary School/High School 7–10	79 (30.6%)	2.28 (1.57, 3.30)	< 0.001	2.20 (1.40, 3.45)	< 0.001
Marital status			< 0.001		0.045
Married/Defacto	74 (16.0%)	Ref.		Ref.	
Single/Widowed/ Separated/Divorced	101 (29.8)	2.23 (1.59, 3.14)		1.52 (1.01, 2.30)	
Household income			0.0051		0.186
Over $100,000	10 (10.2%)	Ref.		Ref.	
$51,000- $100,000	45 (20.7%)	2.30 (1.11, 4.79)	0.026	1.65 (0.73, 3.70)	0.228
< $50,000	79 (27.5%)	3.34 (1.65, 6.75)	< 0.001	2.34 (1.03, 5.32)	0.043
Prefer not to state	41 (20.7%)	2.30 (1.10, 4.81)	0.027	1.68 (0.73, 3.86)	0.220
Anxiety			0.003		0.263
Negative	140 (20.2%)	Ref.		Ref.	
Positive	33 (33.3%)	1.98 (1.25, 3.13)		1.43 (0.76, 2.67)	
Depression			0.008		0.680
Below threshold	141 (20.4%)	Ref.		Ref.	
Above threshold	33 (32.0%)	1.84 (1.17, 2.89)		1.14 (0.61, 2.14)	
Cannabis use			< 0.001		< 0.001
No	122 (17.0%)	Ref.		Ref.	
Yes	52 (67.5%)	10.18 (6.08, 17.04)		6.36 (3.55, 11.37)	
Alcohol consumption			< 0.001		< 0.001
Non Drinker	26 (14.1%)	Ref.		Ref.	
Non- hazardous drinker	25 (13.2%)	0.93 (0.51, 1.67)	0.800	0.93 (0.48, 1.80)	0.831
Hazardous drinker	119 (29.4%)	2.54 (1.60, 4.06)	< 0.001	2.29 (1.34, 3.92)	0.003

^a^Adjusted for age, gender, country of birth, education, marital status, household income, anxiety, depression, cannabis use, and alcohol consumption. This model also included ‘missing’ categories for the following variables: anxiety *n* = 2, depression n = 1, cannabis use n = 1, and alcohol consumption *n* = 5

The odds of being a current smoker were 6.36 times higher among people who used cannabis (*p* < 0.001), compared to non-users. As well, the odds of being a current smoker were 2.29 times higher in people who reported hazardous drinking when compared to those who reported abstaining from alcohol (*p* = 0.003).

## Discussion

Rates of substance use in this population were high, with tobacco smoking and hazardous drinking higher among a sample of orthopaedic trauma patients compared to the general population [7, 39]. Cannabis use and symptoms of anxiety and depression were similar to that found in the general population, [7, 40] but not as high as previously identified in an international sample of the orthopaedic trauma population [11, 41]. Reported rates of drinking, cannabis use, and symptoms of anxiety and depression were found to be significantly higher for current smokers. Further, over a fifth (21.8%) of current tobacco smokers had engaged in comorbid substance use. The study found that males, who reported drinking heavily, had recently used cannabis, had a lower level of educational attainment, and were not in a committed relationship were more likely to be current tobacco users.

The data showing higher comorbid rates of drinking and recent cannabis use among current smokers may warrant addressing these substances together within a single intervention, particularly given that alcohol use has been identified as a predictor of relapse to cigarette smoking [42] and vice versa [43]. Similarly, continued tobacco smoking has been linked to poorer cannabis cessation outcomes. However the impact of cannabis smoking on tobacco cessation is not well explored, [44] and the impact of each other on rates of relapse is not well known [45, 46].

Prochaska et al. [19] found that smoking cessation interventions which address alcohol use also lead to a decrease in other drug use among a sample of polydrug users. Despite this, previous research suggests screening and brief intervention for alcohol use is helpful, however infrequently conducted in the clinical setting [47]. While the current provision of smoking cessation care in hospitals remains sub-optimal despite years of smoke-free policy implementation, [48] a lack of staff time is often reported as being a barrier to the provision of care during hospitalisation [49]. The provision of brief advice for substance use, and screening for anxiety and depression may be time consuming for staff who are already over-burdened. Developing an intervention which builds on current policy and guidelines may facilitate intervention implementation. It may be important to include treatment for symptoms of anxiety and depression when treating tobacco use given the rates of these were found to be significantly higher in current smokers. An online intervention may be one way to increase this provision of care. So too, developing a system to refer individuals to the hospital’s Drug and Alcohol team, or the inclusion of a specialist nurse or counsellor present on ward to assist with addressing these comorbid issues, may provide a solution. However, as with the implementation of any new intervention to change care practices, feasibility, fidelity, and costing of these needs to be determined.

### Strengths and limitations

The relatively high response rate (73%) provides some assurance that this is a representative sample of the orthopaedic trauma population at the two hospitals. Despite this, there may be some limitations with the data as self-report was used to determine rates of substance use and mental ill-health, and may introduce some participant bias to the results, with individual often attaching stigma to the conditions, and sometime unlikely to report rates. For example, as cannabis use is illegal in Australia, it may be that some individuals underreported their use of this substance, with actual rates of use higher than those reported. Given this, these findings may not be generalisable to other orthopaedic trauma populations, with reports of substance use and access to different substances varying from country to country. Further, due to the brevity of the survey, short items were used to determine rates of symptoms of anxiety and depression (however these have previously been found to be reliable) [26, 27]. As well, it is difficult to determine if the rates of anxiety and depression were present before trauma occurred, or were the result of the traumatic event, given the lack of control group. However, that scores were elevated still indicates the need to address these when tackling tobacco in the hospital setting.

## Conclusion

It is important to take into account the relationship between comorbid health conditions when developing health interventions for orthopaedic trauma patients. Given found comorbidities, and that it is known that the current provision of smoking cessation care has previously been found to be low, it is important to take into account the relationship between alcohol, cannabis, and tobacco use when developing interventions for current tobacco users to help them quit smoking while in hospital, and to prevent relapse upon discharge. Further, it is important to acknowledge and incorporate the characteristics of individuals most likely to engage in the target behaviour. One way to do this may be to develop new interventions which integrate existing guidelines to increase the provision of smoking cessation care, but which also incorporate comorbid conditions and user characteristics. Factoring in psychological techniques to promote positive mental health when addressing tobacco cessation in this population should also be a priority. Addressing these health behaviours may not only promote a faster recovery from surgery, but may also prevent readmission and therefore the cost to the health care system. Health behaviour interventions addressing smoking, substance use and mental ill-health are warranted for the orthopaedic trauma population.

## Additional files

Additional file 1: Supplement 1. Title of data: Survey items, Description of data: Lists all survey items, response options and the references for these. (DOCX 24 kb)

Additional file 2: Supplement 2. Title of data: Cronbach’s alpha statistics, Description of data: Cronbach’s alpha statistics for established survey. (DOCX 45 kb)

## Abbreviations

AUDIT-C: Alcohol Use Disorder Identification Test
CI: Confidence Interval
GAD-2: Generalised Anxiety Disorder two item
NRT: Nicotine Replacement Therapy
OR: Odds Ratio
OTI: Opiate Treatment Index
PHQ-2: Patient Health Questionnaire
SD: Standard Deviation
