# Supplementary material for: Comorbid tobacco and other substance use and symptoms of anxiety and depression among hospitalised orthopaedic trauma patients
Source: BMC Psychiatry. 2019 Jan 17;19:28. doi: 10.1186/s12888-019-2021-y (PMC6337866; doi:10.1186/s12888-019-2021-y)
Supplement: Supplementary file 1 — Supplement 1. Title of data: Survey items, Description of data: Lists all survey items, response options and the references for these. (DOCX 24 kb) [file 12888_2019_2021_MOESM1_ESM.docx]

**Supplement 1.** Survey items

| **Survey question** | **Response options** |
| --- | --- |
| Smoking status and smoking related variables^1-6^ | |
| Do you currently smoke tobacco | Yes, daily |
|  | Yes, at least once a week |
|  | Yes, less than once a week |
|  | No, not at all |
| Have you smoked at least 100 cigarettes or a similar amount of tobacco in your life? | Yes |
|  | No |
|  | Not sure |
| AUDIT-C Alcohol use^7-9^ | |
| How often did you have a drink containing alcohol in the past year? | Never |
|  | Monthly or less |
|  | 2-4 times a month |
|  | 2-3 times a week |
|  | 4 or more times a week |
| How many standard drinks containing alcohol do you have on a typical day? | 0 drinks |
|  | 1 or 2 drinks |
|  | 3 or 4 drinks |
|  | 5 or 6 drinks |
|  | 7 to 9 drinks |
|  | 10 or more drinks |
| How often have you had four or more standard drinks on one occasion? | Never |
|  | Less than monthly |
|  | Monthly |
|  | Weekly |
|  | Daily or almost daily |
| Cannabis Use^10^ | |
| Have you used cannabis (marijuana, dope, grass, hash, pot) in the last 30 days | Yes |
|  | No |
| Do you ever mix tobacco with cannabis (marijuana, dope, grass, hash, pot)? | Yes, every time I smoke |
|  | Yes, sometimes |
|  | No, never |
| GAD-2 Anxiety symptoms*^11^ | |
| Over the last 2 weeks, how often have you been bothered by the following problem? | Feeling nervous, anxious or on edge |
|  | Not being able to sleep or control worrying |
| PHQ-2 Depression symptoms*^12^ | |
| Over the last 2 weeks, how often have you been bothered by any of the following problems? | Little interest or pleasure in doing things |
|  | Feeling down, depressed, or hopeless |

* on a four point Likert scale (not at all; several days; more than half the days; nearly every day).

**References**

1. Heatherton TF, Kozlowski LT, Frecker RC, Rickert W, Robinson J. Measuring the heaviness of smoking: using self‐reported time to the first cigarette of the day and number of cigarettes smoked per day. *Br J Addict*. 1989;**84**(7):791-800.

2. Guillaumier A, Bonevski B, Paul C, D'Este C, Doran C, Siahpush M. Paying the price: a cross-sectional survey of Australian socioeconomically disadvantaged smokers' responses to hypothetical cigarette price rises. *Drug Alcohol Rev*. 2014;**33**(2):177-85.

3. Mullins R, Borland R. Changing the way smoking is measured among Australian adults: a preliminary investigation of Victorian data. *Quit evaluation studies*. 91998. p. 163-73.

4. Lacchetti C, Cohen J, Ashley MJ, Ferrence R, Bull S, de Groh M, Pederson L. Is nicotine dependence related to smokers' support for restrictions on smoking? *Nicotine Tob Res*. 2001;**3**(3):257-60.

5. John U, Meyer C, Schumann A, Hapke U, Rumpf HJ, Adam C, Alte D, Ludemann J. A short form of the Fagerstrom Test for Nicotine Dependence and the Heaviness of Smoking Index in two adult population samples. *Addict Behav*. 2004;**29**(6):1207-12.

6. Heatherton TF, Kozlowski LT, Frecker RC, Fagerstrom KO. The Fagerstrom Test for Nicotine Dependence: a revision of the Fagerstrom Tolerance Questionnaire. *Br J Addict*. 1991;**86**(9):1119-27.

7. Bush K, Kivlahan DR, McDonell MB, Fihn SD, Bradley KA. The AUDIT alcohol consumption questions (AUDIT-C): an effective brief screening test for problem drinking. Ambulatory Care Quality Improvement Project (ACQUIP). Alcohol Use Disorders Identification Test. *Arch Intern Med*. 1998;**158**(16):1789-95.

8. Bradley KA, Bush KR, Epler AJ, Dobie DJ, Davis TM, Sporleder JL, Maynard C, Burman ML, Kivlahan DR. Two brief alcohol-screening tests From the Alcohol Use Disorders Identification Test (AUDIT): validation in a female Veterans Affairs patient population. *Arch Intern Med*. 2003;**163**(7):821-9.

9. Bradley KA, DeBenedetti AF, Volk RJ, Williams EC, Frank D, Kivlahan DR. AUDIT‐C as a brief screen for alcohol misuse in primary care. *Alcoholism, clinical and experimental research*. 2007;**31**(7):1208-17.

10. Darke S, Ward J, Hall W, Heather N, A W. The Opiate Treatment Index (OTI) Researcher’s Manual. Sydney: National Drug and Alcohol Research Centre; 1991. Contract No.: 11.

11. In Q. The 2-item Generalized Anxiety Disorder scale had high sensitivity and specificity for detecting GAD in primary care. *Intern Med*. 2007;**146**:317-25.

12. Lowe B, Kroenke K, Grafe K. Detecting and monitoring depression with a two-item questionnaire (PHQ-2). *J Psychosom Res*. 2005;**58**(2):163-71.
