# Supplementary figures and images for: Comorbid tobacco and other substance use and symptoms of anxiety and depression among hospitalised orthopaedic trauma patients
Source: BMC Psychiatry. 2019 Jan 17;19:28. doi: 10.1186/s12888-019-2021-y (PMC6337866; doi:10.1186/s12888-019-2021-y)

**AUDIT- C score**


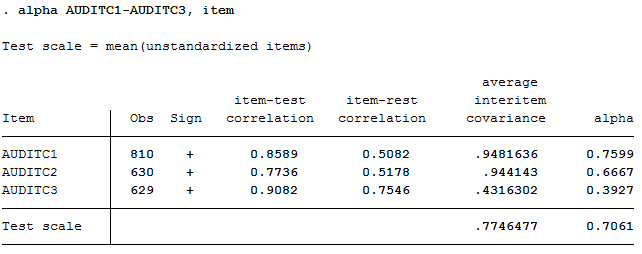


**PHQ-2**


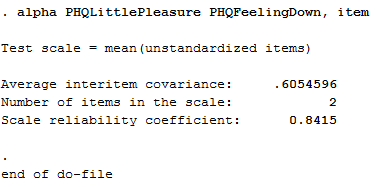


**GAD-2**


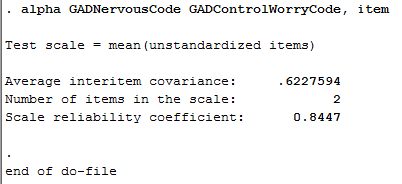

Supplement: Supplementary file 2 — Supplement 2. Title of data: Cronbach’s alpha statistics, Description of data: Cronbach’s alpha statistics for established survey. (DOCX 45 kb) [file 12888_2019_2021_MOESM2_ESM.docx]
